# Metabolomic Insights into the Allelopathic Effects of *Ailanthus altissima* (Mill.) Swingle Volatile Organic Compounds on the Germination Process of *Bidens pilosa* (L.)

**DOI:** 10.3390/metabo15010012

**Published:** 2025-01-03

**Authors:** Leonardo Bruno, Diana M. Mircea, Fabrizio Araniti

**Affiliations:** 1 Department of Biology, Ecology, and Hearth Sciences (DiBEST), University of Calabria, 87036 Arcavacata, Italy; leonardo.bruno@unical.it; 2 Mediterranean Agroforestry Institute (IAM), Universitat Politècnica de València, Camino de Vera s/n, 46022 Valencia, Spain; dmmircea@upvnet.upv.es; 3 Department of Agricultural and Environmental Sciences—Production, Landscape, Agroenergy, University of Milan, 20133 Milan, Italy

**Keywords:** allelopathy, alien species, metabolomics, plant metabolism, phytotoxicity

## Abstract

**Background/Objectives:** This study explores the allelopathic effects of volatile organic compounds (VOCs) emitted by the invasive species *Ailanthus altissima* (Mill.) Swingle on the seed germination of *Bidens pilosa*. *A. altissima* is known for releasing allelopathic VOCs that suppress the growth of neighbouring plants, contributing to its invasive potential. **Methods:** To examine these effects, we exposed *B. pilosa* seeds to varying concentrations of *A. altissima* VOCs, assessing germination rates and metabolic changes through untargeted metabolomics. **Results:** Our findings revealed that VOCs from *A. altissima* significantly inhibited the germination speed and overall germination rates of *B. pilosa* in a dose-dependent manner. Metabolomic profiling showed disruptions in energy and amino acid metabolism pathways, specifically involving delayed breakdown of starch and key metabolites, indicating inhibition of critical metabolic processes during early germination stages. This metabolic delay likely impairs *B. pilosa*’s establishment and competitiveness, enhancing *A. altissima*’s ecological dominance. **Conclusions:** The results underscore the potential of VOC-based allelopathy as a mechanism of plant invasion, offering insights into the role of VOCs in interspecies plant competition and ecosystem dynamics.

## 1. Introduction

Plant–plant communication through volatile organic compounds (VOCs) represents a fascinating area of research that elucidates how plants can influence one another’s growth and defence mechanisms [1]. This communication often occurs when neighbouring plants release VOCs in response to herbivore attacks or other stressors, which can prime adjacent plants to enhance their defensive strategies [2].

Plant volatiles are chemicals that give plants a characteristic and typical odour, flavour, and several other properties. They are complex mixtures mainly composed of terpenes, synthesised within plants as secondary metabolites, providing plants with many ecological advantages [3]. They play a pivotal role in plant reproduction, acting as pollinator attractants, providing protection against herbivores, and mediating plant communication, being determinants for vegetation patterning [3,4]. These chemicals are thought to be important allelopathic agents, especially in arid and semi-arid conditions where they act in the vapour phase [5,6,7], and early studies demonstrated that plant volatiles are potent seed germination inhibitors, playing an important role in ecosystems by reducing seedling establishment ability and growth [6,8,9]. In addition, several studies demonstrated that specific volatile compounds can significantly impede seed germination rates. For instance, Kang et al. [10] identified L-Fenchone and 1,8-Cineole from the leaves of Star Anise (*Illicium verum*), which exhibited notable allelopathic activity when tested on lettuce seeds using the Dish pack method. Their findings indicated that these volatiles could effectively inhibit germination, highlighting the potential of using such compounds to manage weed populations [10]. Similarly, Mishyna et al. [11] reported that octanal, a compound released from *Heracleum sosnowskyi* fruit, strongly inhibited both seed germination and radicle elongation in lettuce, suggesting that octanal is a key contributor to the allelopathic effects of this species [11].

The mechanism by which these volatiles exert their inhibitory effects often involves interference with physiological processes in the target plants. Maffei et al. [12] noted that certain monoterpenes, such as carvacrol and D-limonene, can disrupt the cytochromic respiration pathway and inhibit growth and seed germination in neighbouring plants. Also, the delay of seed germination and growth is an important ecological effect, which plays a significant ecological role in natural ecosystems, increasing the competitive ability of the species, which can retard the growth of the competitors. McCalla and Norstadt [13] defined germination as the most sensitive vegetative stage to phytotoxins. A short period of inhibition or stimulation at this stage could strongly increase or reduce the ability to compete with other plants [13,14].

One worldwide-spread species known for its strong allelopathic properties is *Ailanthus altissima* (Mill.) Swingle, the “tree of heaven”. Originally from Asia, *A. altissima* has become a widespread invasive species in many parts of the world, often dominating native ecosystems [15]. Its allelopathic effects have been well documented, with studies revealing its ability to inhibit the growth and germination of various plant species, thereby contributing to its competitive advantage in non-native regions [16,17]. The VOCs emitted by *A. altissima* could be of particular interest, as they may play a role in its invasive success by inhibiting or altering the growth patterns of neighbouring plants. While *A. altissima*’s general allelopathic properties are recognised, there is limited research on the specific effects of its VOCs on the germination process of neighbouring species. This represents a notable gap in the study of plant–plant chemical interactions. Given the potential role of VOCs in delaying or inhibiting germination, research into their specific impact could contribute valuable insights into the invasiveness ability of *A. altissima*.

One neighbouring species of interest is *Bidens pilosa* L. (Asteraceae), commonly known as blackjack. Originally from Central and South America, this fast-growing weed is now widely distributed across tropical and subtropical regions, posing a threat to vital crops [18]. Several traits contribute to the invasiveness of *B. pilosa*. It exhibits a high growth rate, capable of producing multiple generations annually, and generates an abundant number of seeds. These seeds readily attach to animal fur, bird feathers, and human clothing, facilitating its spread to new areas. The seeds germinate rapidly under diverse conditions, enhancing the plant’s adaptability. *B. pilosa* thrives in a wide range of environmental settings [19].

Renowned for its aggressive colonisation, prolific seed production, and adaptability to disturbed habitats, *B. pilosa* presents a significant challenge to agriculture and native ecosystems. Its allelopathic potential further strengthens its competitive edge, making it an excellent model for studying plant–plant interactions [20]. Investigating the effects of *Ailanthus altissima*’s volatile organic compounds (VOCs) on this invasive weed could provide valuable insights into the ecological impacts of allelopathic interactions and their role in the success of invasive species.

The present study aims to investigate the allelopathic potential of VOCs emitted by *A. altissima* on the germination of *B. pilosa*. By examining how these VOCs influence the metabolic activity and germination timing of *B. pilosa* seeds, this research seeks to demonstrate the potential of VOC-based allelopathy as a natural means of plant invasion. Our findings reveal that the VOCs released by *A. altissima* slow down the germination metabolism of *B. pilosa*, delaying its germination and potentially reducing seedlings’ establishment and competitiveness.

## 2. Materials and Methods

### 2.1. Plant Material

Aerial parts of *Ailanthus altissima* were sampled in June in Calabria (Southern Italy). The plant material was collected in an invaded ecosystem close to Reggio Calabria (Italy) (latitude N 38°16′9030″, longitude E 15°67′5358″), and its collection did not require any specific permission.

### 2.2. Germination Indexes

To allow for the synchronisation of seed germination, the seeds of *Bidens Pilosa* were poured into a beaker filled with deionised water and stored for two days in a refrigerator at 4 °C. The seeds were then successively blotted on filter paper and dried at room temperature under a chemical cabin with laminar flow. Once dried, the seeds were sterilised for 15 min in 5% NaClO (Merk Life Science, Milan, Italy) solution, and 20 seeds (for each replicate and concentration) were sown in Petri dishes (9 cm in Ø), with the bottom covered by a double layer of filter paper and 5 mL of deionised water.

To verify if the VOCs released by the leaves of *A. altissima* could inhibit seed germination, the Petri dishes (without the lids) were exposed for 8 days in an open box to different amounts (0, 50, 100, 150, 200, and 250 g) of freshly collected *A. altissima* leaves (changed daily) following the protocol previously described by Araniti et al. [6].

The germination was monitored daily, and then the germination index [G_T_(%)] and speed of germination (S) were calculated using the following formula:

G_T_(%) = [(NT × 100)/N]

where N_T_ is the number of germinated seeds and N is the number of seeds used in the bioassay.

S = (N_1_ × 1) + (N_2_ − N_1_) × 1/2

N_1_, N_2_, N_3_, …… N_n−1_, N_n_; the proportion of the germinated seed obtained at the first (1), the second (2), the third (3), (*n* − 1), n hours or days after sowing.

This experiment allowed for us to determine the key concentration of 150 g of fresh material, which was then used for the metabolomic analysis.

### 2.3. VOC Characterisation: HS-SPME-GC–MS Analysis

VOC characterisation was carried out through an HS-SPME-GC–MS analysis using a gas chromatographer (Agilent 7890A GC—Agilent Scientific Instruments, Santa Clara, CA, USA) coupled with a single quadrupole mass spectrometer (Agilent 5975C—Agilent Scientific Instruments, Santa Clara, CA, USA) and equipped with a MEGA-5MS capillary column (30 m × 0.25 mm × 0.25 µm plus 10 m pre-column) (MEGA S.r.l., Legnano (MI), Italy). Plant material (1 g per replicate) was incubated for 20 min in a 20 mL glass vial at room temperature. Subsequently, an SPME grey fibre (StableFlex, coated with divinylbenzene/Carboxen on polydimethylsiloxane; 50/30 μm coating; Merk Life Science, Milan, Italy) was exposed to the volatile organic compounds (VOCs) released by the plants for 20 min to allow adsorption onto the fibre. The adsorbed sample was then desorbed into the GC–MS, operating in splitless mode. The injector was settled at 200 °C, and the transfer line was set at 250 °C, whereas the source and the quadrupole were set at 280 °C and 150 °C, respectively. The temperature program was set as follows: an isocratic hold at 45 °C for 7 min, followed by a temperature increase from 45 °C to 80 °C at a rate of 10 °C per minute, then from 80 °C to 200 °C at a rate of 20 °C per minute, and a final isocratic hold at 200 °C for 3 min.

### 2.4. Untargeted Metabolomic Analysis

To evaluate the effects of coumarin on *B. pilosa* seed metabolism during the germination process, seeds were treated as previously described using 150 g of *A. altissima* leaves. This concentration was chosen since the higher concentration did not reduce total germination but significantly reduced its speed.

Seeds were collected at different time points (T0 = day 0; T1 = day 2; T2 = day 4; T3 = day 6; T4 = day 8), snap-frozen in liquid nitrogen, and powdered. A total of 100 mg of plant material for each treatment (0 g and 150 g) replicate was transferred into a 2 mL vial.

Extraction and sample derivatisation were carried out following the protocol proposed by Lisec et al. [21] and modified by Araniti et al. [22].

For extraction, 1400 μL of chilled methanol (−20 °C) was added along with 60 μL of ribitol (0.2 mg/mL in water) as an internal standard for the polar phase, followed by vortexing for 10 s. The tubes were heated in a thermomixer at 70 °C with shaking at 950 rpm for 10 min and then centrifuged at 11,000 g for 10 min. The resulting supernatants were transferred to glass vials, where 750 μL of chilled chloroform (−20 °C) and 1500 μL of cold water (4 °C) were added sequentially. After vortexing for 10 s, the samples were centrifuged again at 2200 g for 15 min.

The polar phase (150 μL) from each sample was collected into 1.5 mL tubes and dried using a vacuum concentrator without heating.

For derivatisation, 40 μL of methoxyamine hydrochloride (20 mg/mL in pyridine) (Merk Life Science, Milan, Italy) was added to the dried samples, which were incubated in a thermomixer at 37 °C and shaken at 950 rpm for 2 h. This step was followed by silylation, achieved by adding 70 μL of MSTFA (Merk Life Science, Milan, Italy) to each sample and shaking at 37 °C for 30 min. Finally, 110 μL of the derivatised samples was transferred to GC/MS-compatible glass vials for analysis.

The derivatised extracts were analysed using a MEGA-5MS capillary column (30 m × 0.25 mm × 0.25 µm, equipped with a 10 m pre-column) in a gas chromatography system (Agilent 7890A GC) coupled with a single quadrupole mass spectrometer (Agilent 5975C). The injector and ion source temperatures were set to 250 °C and 260 °C, respectively. A 1 µL aliquot of each sample was injected in splitless mode, with helium as the carrier gas at a 1 mL/min flow rate. The temperature program included an initial isothermal period at 70 °C for 5 min, followed by a temperature increase of 5 °C/min to 350 °C, and a final hold at 330 °C for 5 min. Mass spectra were acquired in electron ionisation (EI) mode at 70 eV, scanning from 40 to 600 *m*/*z* with a scan time of 0.2 s. A solvent delay of 9 min was applied. Samples, n-alkane standards, and blank pyridine solvent were periodically injected to assess instrument performance, confirm tentative identifications, and track any retention index (RI) shifts. Solvent blanks were run between samples to monitor potential contamination, and each mass was verified against the blanks to rule out contamination sources.

### 2.5. GC/MS Data Analysis Using MS-DIAL

Raw peak extraction in this study was conducted using MS-DIAL with an open-source, publicly available EI spectral library. The baseline of the data was filtered and calibrated, and subsequent steps—such as peak alignment, deconvolution analysis, peak identification, and peak height integration—were performed following the methodology outlined in Misra et al. [23]. Detection parameters included an average peak width of 20 scans, a minimum peak height threshold of 1000 amplitudes, and a sigma window value of 0.5, with an EI spectra cut-off set at 5000 amplitudes for deconvolution. The identification of peaks utilised a retention time tolerance of 0.2 min, an *m*/*z* tolerance of 0.5 Da, an EI similarity threshold of 60%, and a minimum identification score of 80%. Alignment parameters were adjusted with a retention time tolerance of 0.5 min and a retention time factor of 0.5.

For MS-DIAL data annotations, compound identification was achieved using a homemade library built following the protocol proposed by Misra [24].

Only confidently annotated and quantified metabolites were reported. Metabolite identification followed the Metabolomics Standards Initiative (MSI) guidelines [25]: Level 2 identification was based on spectral database matching with a match factor above 80%; Level 3 allowed for compound group identification through specific ions and retention time regions.

Concerning VOC data, all the putatively annotated metabolites resulting from MS-DIAL to have a total score higher than 80% were checked one-by-one on several open-source plant databases (www.plantcyc.org, accessed on 1 October 2024) and/or by checking in the literature if these molecules were previously annotated on the same genus or species. Those metabolites not belonging to the plant metabolome were considered unknowns and removed from the analysis.

### 2.6. Statistical Analysis

The experiments were conducted using a randomised design with five replications for the germination tests and four replications for metabolomics experiments. Germination parameters were previously tested for normality and homogeneity of the variance and successively analysed through one-way ANOVA using the LSD test as post hoc (*p* ≤ 0.05).

Metabolomic data were analysed using Metaboanalyst 6.0 [26]. Using the R-based open-source software MetaboAnalyst 6.0, data were normalised using the internal standard (ribitol—0.02 mg/mL) to account for variations in sample preparation, instrument performance, and extraction efficiency, ensuring consistency and comparability across samples, and the missing values were replaced with half of the minimum value in the data set. Successively, data were Log_10_ transformed to reduce the impact of large differences in metabolite concentrations, making the data more manageable and highlighting relative changes rather than absolute differences. Finally, transformed data were Pareto scaled (mean-centred and divided by the square root of the standard deviation of each variable) to further enhance this by reducing the dominance of highly abundant metabolites while still preserving the data’s structure, making subtle variations more apparent and improving the interpretability of multivariate analyses.

Data were then classified through principal component analysis (PCA) to provide an overview of the quality of the data acquisition step and evaluate group separation. Furthermore, cluster analysis was performed using the Euclidean distance measure and the Ward algorithm for cluster formation to examine the classification further.

A within-subjects two-way ANOVA was used, and the significance threshold was defined as the corrected *p*-value ≤ 0.05. The false discovery rate was chosen for multiple testing corrections. Moreover, GC–MS data were analysed using Multivariate Empirical Bayes Analysis of Variance (MEBA) for Time Series, and features with significant changes over time (using the Hotelling-T2 values) were examined for their dose–response during the germination process.

## 3. Results

### 3.1. Germination Index

**Figure 1:**
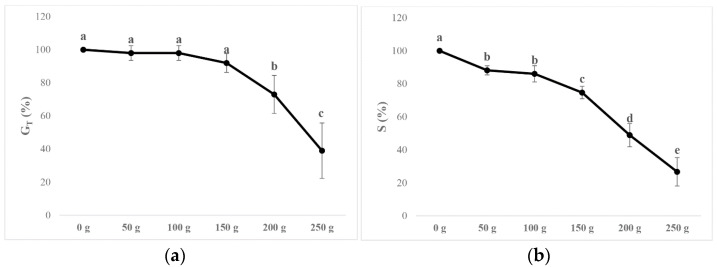
Dose-response curves of the (**a**) germination index [GT(%)] and (**b**) germination speed (S) of *B. pilosa* seeds exposed for 8 days to *Ailanthus altissima* VOCs. Data are expressed as mean ± SD and analysed through one-way ANOVA using the LSD test as post hoc (*p* ≤ 0.05). Different letters along the curves (a–e) indicate statistical differences among the treatments with *p* ≤ 0.05. N = 4.

The results revealed a significant inhibition potential of *A. altisisma* VOCs on *B. pilosa* seed germination. In particular, the chemicals released from the plant material significantly reduced total germination (G_T_ %) and speed (S %). The S parameter was significantly inhibited (≈12%), even at the lower treatments (50 g and 100 g), increasing up to 73% at the highest concentration assayed (250 g of plant material) (Figure 1). Conversely, the lowest treatments (from 50 g to 150g) did not affect the GT parameter. The 200 g treatment caused 27% inhibition, reaching 60% at the highest concentration (Figure 1). It should be noted that, enlarging the end of the experiments to 13 days, almost all seeds were germinating, even at 250 g treatment.

### 3.2. VOC Characterisation

Table 1 presents a chemical breakdown of the volatile organic compounds (VOCs) derived from *Ailanthus altissima.* The major chemical classes identified include alcohols, polyols, carbonyl compounds, fatty acid esters, alkanes, and monoterpenoids (Table 1).

The Relative Area Percentage (RAP%) column quantifies the abundance of each compound within the sample, offering insights into which chemicals dominate the volatile profile of *Ailanthus altissima*. The most abundant compound identified is (Z)-3-hexen-1-ol, a fatty alcohol comprising 11.09% of the total volatile composition (Table 1). This compound, alongside 3-hexenal (10.18%) and hexyl trifluoroacetate (9.46%), suggests a volatile profile rich in fatty alcohols and esters (Table 1). Alcohols and polyols form a significant portion of the volatile makeup, with notable entries like 2-methyl-1-butanol (8.33%) and 1-pentanol (5%) (Table 1). Carbonyl compounds, such as 3-hexenal and hexanal, are similarly well represented. Meanwhile, unsaturated aliphatic hydrocarbons (e.g., (Z)-3-octene, at 8.88%) and monoterpenoids (compounds like *β*-thujene and *α*-pinene, albeit at lower concentrations) add another layer to the VOC profile (Table 1). In summary, the chemical characterisation of *A. altissima* reveals a rich blend of volatile compounds, dominated by fatty alcohols, carbonyl compounds, and alcohols.

### 3.3. GC–MS Untargeted Metabolomics

A GC/MS-driven untargeted metabolic analysis was conducted to gain more insights into the *A. altissima* VOC-mediated metabolic changes induced in *B. pilosa* seed metabolism during germination.

The GC–MS-driven analysis was performed on seeds exposed to 150 g of freshly cut leaves of *A. altissima* for several days (T0 = day 0; T1 = day 2; T2 = day 4; T3 = day 6; T4 = day 8). The analysis revealed grouped and individual metabolites that allowed for sample discrimination. Among all the analysed stages, the metabolomic analysis allowed for us to annotate and quantify 61 metabolites, mainly belonging to the classes of amino acids, organic acids, sugars, and sugar alcohols, among others (Supplementary Table S1).

**Figure 2:**
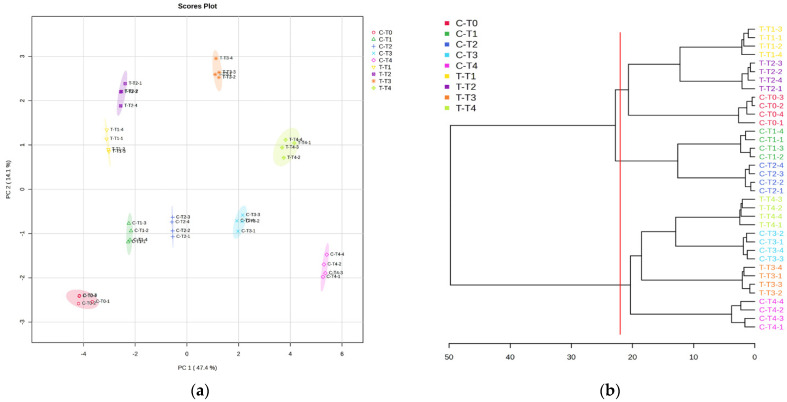
Effects of *A. altissima* VOCs on *Bidens pilosa* seed metabolome. (**a**) Unsupervised PCA scores plot between the two selected PCs (the explained variances are shown in brackets); (**b**) Clustering result shown as a dendrogram (distance measure using Euclidean, and clustering algorithm using ward). N = 4.

After sample normalisation, transformation, and scaling, the data were statistically analysed through unsupervised principal component analysis (PCA) to obtain a global view of the kinetic metabolic patterns of developing *B. pilosa* seeds during VOC exposition. The PCA was built by virtue of the first two components (PC1 vs. PC2), which accounted for 61.5% of the total variance (Figure 2).

The PCA analysis provides a detailed perspective on the time-dependent effects of Ailanthus altissima volatiles (VOCs) on seeds, revealing distinct patterns of variation between the treated and untreated groups (Figure 2). The analysis highlights the role of PC1 in capturing temporal variation, while PC2 primarily differentiates seeds based on the treatment, i.e., the presence or absence of VOC exposure (Figure 2).

The progression along PC1, which explains 47.4% of the total variance, is largely driven by the time-related dynamics in both treated and untreated seeds. For untreated seeds (C-T0 through C-T4), there is a clear temporal progression from left to right along PC1. This trend suggests that, even in the absence of VOCs, the seeds undergo gradual physiological changes over time, likely reflecting natural developmental processes. Similarly, treated seeds (T-T1 through T-T4) show a temporal trend along PC1 but with a much more pronounced shift, indicating that exposure to VOCs accelerates or amplifies time-dependent changes in seed physiology (Figure 2).

In contrast, PC2, which accounts for 14.1% of the variance, serves as the axis of differentiation between the treatment groups. Untreated seeds cluster together with minimal dispersion along PC2, reflecting the uniformity of their responses in the absence of VOCs. Treated seeds, however, are clearly separated from untreated seeds along this axis, demonstrating the substantial impact of VOC exposure. This separation is consistent across all time points, indicating that VOCs introduce a distinct set of changes in seed responses that are independent of natural temporal variation (Figure 2).

Interestingly, the combined interpretation of PC1 and PC2 reveals that the effects of VOCs become increasingly pronounced over time. At the initial stages (T-T1), the treated seeds are still relatively close to the untreated groups along PC1, indicating that time-related changes are just beginning to manifest. However, as time progresses, the treated seeds move further along PC1, with significant dispersion along PC2 as well. This suggests that, while PC1 captures the cumulative, time-dependent effects of VOCs, PC2 highlights the specific impact of the treatment itself, which becomes more variable and pronounced with longer exposure (Figure 2).

By T-T4, the treated seeds form a distinct and isolated cluster, far removed from the untreated seeds on both axes. This reinforces the idea that the VOCs not only accelerate time-dependent changes (as seen along PC1) but also introduce unique responses that are absent in the untreated seeds (as captured by PC2). The lack of overlap between the treated and untreated clusters at all time points highlights the robustness of the VOC effect (Figure 2).

The evaluation of the PCA loading plots pointed out that PC1 was positively influenced by glutamine, sorbose, ribose, fructose, isoleucine, serine, and proline, whereas melezitose and raffinose, methylmalonic acid, and galactinol negatively influenced it. On the contrary, PC2 was positively dominated by proline, maltose, putrescine, 4-hydroxybutiric acid, and glucose, whereas 4-aminobutyric acid, sorbose, glyceric acid, succinic acid, asparagine, and norleucine, among others, negatively affected it (Supplementary Table S1).

The separation trend observed during the unsupervised PCA was further investigated by the cluster analysis, which highlighted the formation of three main groups (Figure 2). The first group was formed by the control seeds at T0 (C-T0) and the treated seeds at T1 and T2 (T-T1 and T-T2). The second group was formed only by the controls at T1 and T2 (C-T1 and C-T2), whereas the remaining treatments formed the third group (C-T3, C-T4, T-T3, and T-T4) (Figure 2).

**Figure 3:**
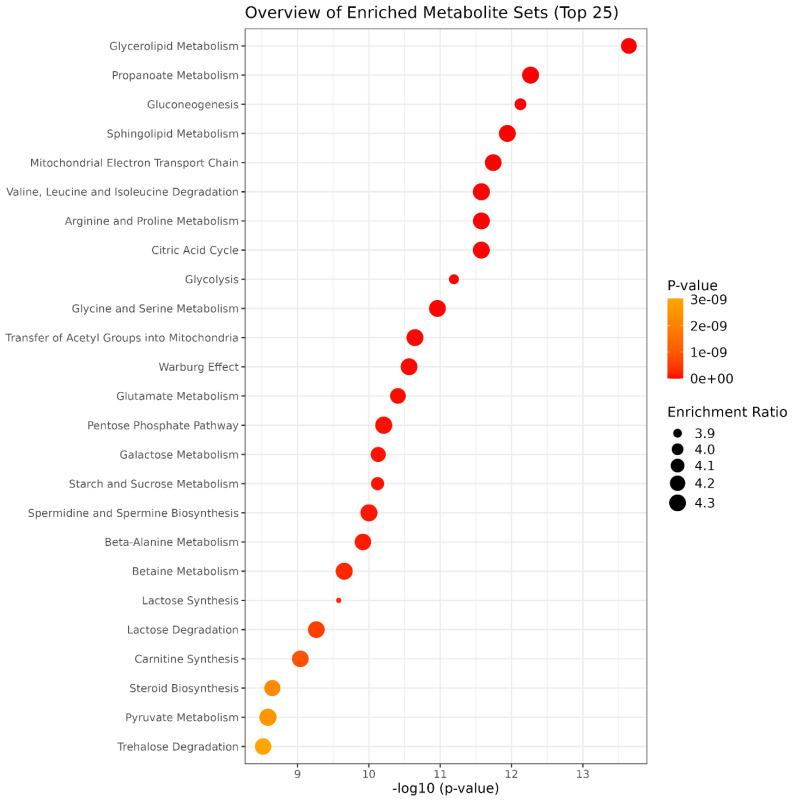
Pathway enrichment analysis revealed different metabolic pathways enriched during *Bidens pilosa* germination in response to different coumarin doses (0–800 µM). (*p*-value cut off ≤ 0.05).

A KEGG-based enrichment analysis, performed to identify the classes of metabolites that were overrepresented in a large set of metabolites, revealed enrichment of glycerolipid and propanoate metabolism; gluconeogenesis; sphingolipid metabolism; mitochondrial electron transport chain; valine, leucine, and isoleucine degradation; arginine and proline metabolism; citric acid cycle; and glycolysis, among others (Figure 3 and Supplementary Table S1).

Successively, data were analysed through pathway analysis, which combines enrichment and topology analysis. It pointed out that 30 pathways (14 with an impact higher > 0.2) were significantly changed during seed germination, and those more affected were involved in amino acid and sugar metabolism (Table 2 and Supplementary Table S1).

**Figure 4:**
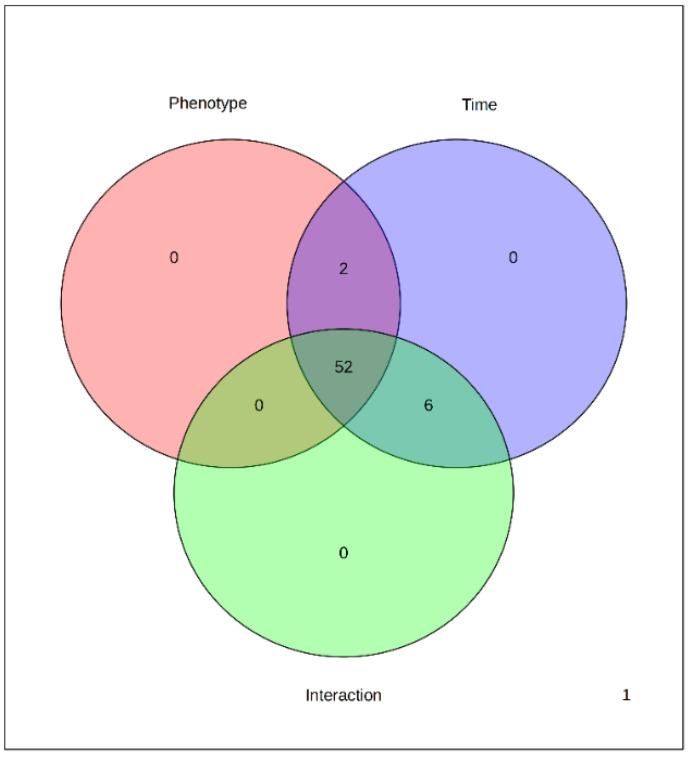
Venn diagram summary of results from two-way ANOVA.

A two-way ANOVA was then conducted to determine which factors (developmental time, VOC treatment, and their interaction) cause the variation in each metabolite. Among the identified 60 metabolites, 54 were influenced by the VOCs (all excluded glucose-1-phosphate, trisaccharide, 4-aminobutyric acid, sinigrin, salicyl alcohol-b-glucoside, stearic acid, melezitose), all were influenced by the time, and 58 were influenced by the interaction between time and treatment (all metabolites excluded norleucine and 1,6-anhydroglucose) (Figure 4 and Supplementary Table S1).

The multifactorial two-way ANOVA confirmed the significance of the data, which were further analysed through Multivariate Empirical Bayes Analysis of Variance (MEBA) for Time Series, a statistical approach commonly used to identify and analyse temporal patterns across multiple variables within time series data. The approach is designed to compare the time-course profiles under different conditions. The result is a list of variables ranked by their differences in temporal profiles across different biological conditions. The Hotelling-T2 was used to rank the variables with different temporal profiles between the biological conditions under study.

**Figure 5:**
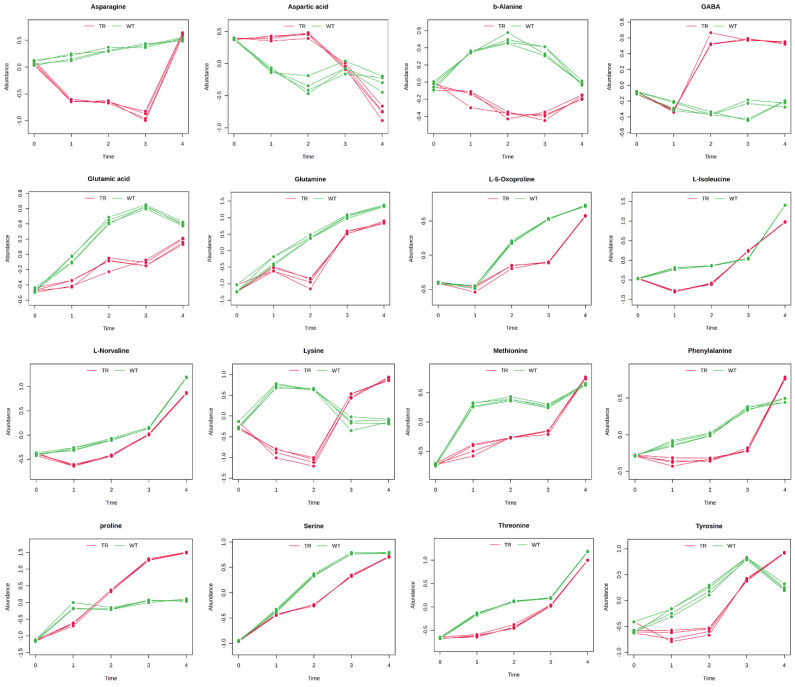
Amino acids with distinctive temporal profiles between control plants (WT) and plants exposed to *A. altissima* VOCs (TR). The temporal profiles are presented as line graphs based on the two conditions under study: green for the WT and red for the TR. The graphs represent the normalised relative abundance profiles of the amino acids with a good classification score and previously significantly affected by the two-way ANOVA (*p* ≤ 0.05). N = 4.

**Figure 6:**
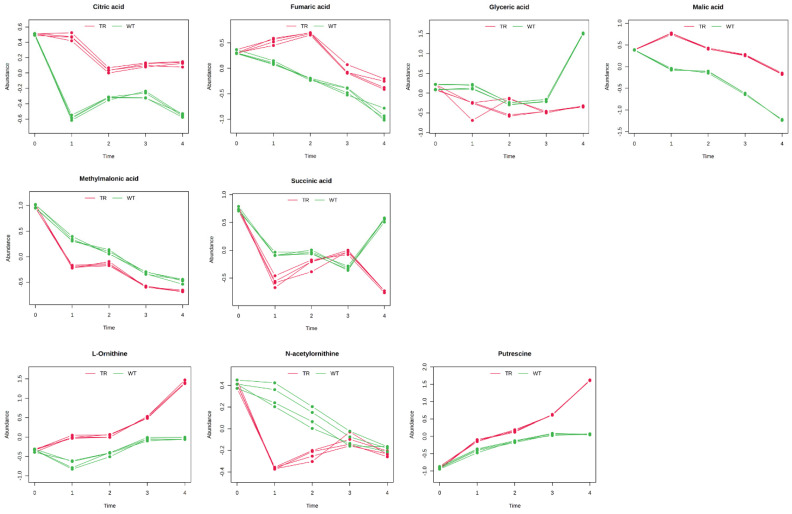
Organic acids and polyamines with distinctive temporal profiles between control plants (WT) and plants exposed to *A. altissima* VOCs (TR). The temporal profiles are presented as line graphs based on the two conditions under study: green for the WT and red for the TR. The graphs represent the normalised relative abundance profiles of the organic acids and polyamines with a good classification score and previously resulted significantly affected by the two-way ANOVA (*p* ≤ 0.05). N = 4.

**Figure 7:**
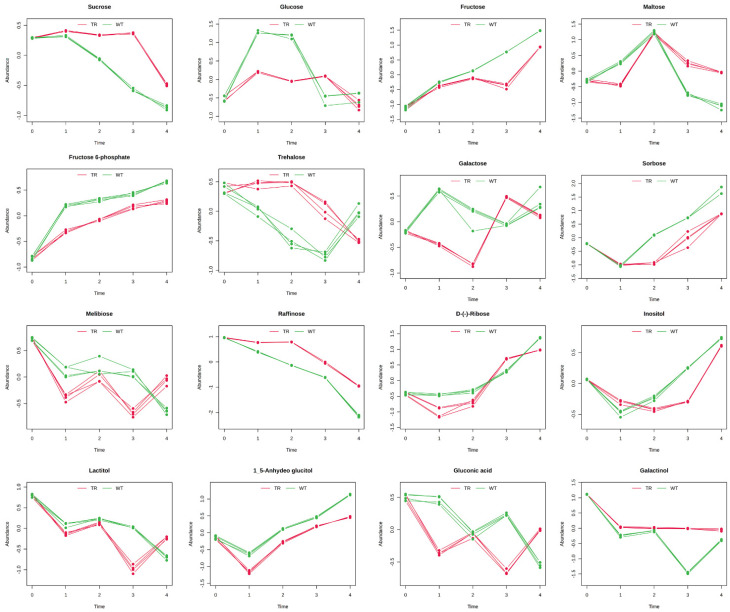
Sugars with distinctive temporal profiles between control plants (WT) and plants exposed to *A. altissima* VOCs (TR). The temporal profiles are presented as line graphs based on the two conditions under study: green for the WT and red for the TR. The graphs represent the normalised relative abundance profiles of the sugars with a good classification score and previously resulted significantly affected by the two-way ANOVA (*p* ≤ 0.05). N = 4.

**Figure 8:**
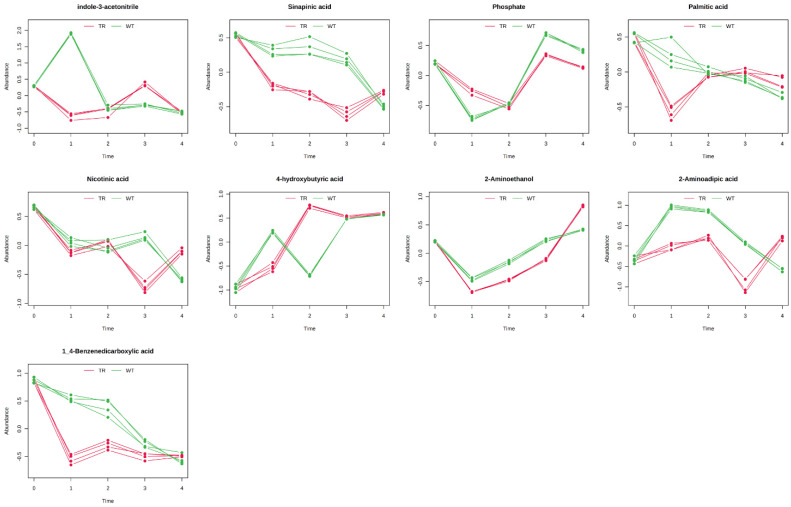
Miscellaneous (molecules belonging to different classes) with distinctive temporal profiles between control plants (WT) and plants exposed to *A. altissima* VOCs (TR). The temporal profiles are presented as line graphs based on the two conditions under study: green for the WT and red for the TR. The graphs represent the normalised relative abundance profiles of the different classes of compounds with a good classification score and previously resulted significantly affected by the two-way ANOVA (*p* ≤ 0.05). N = 4.

The results were grouped into chemical classes (amino acids, Figure 5; organic acids and polyamine, Figure 6; sugars, Figure 7; miscellanea, Figure 8) and graphically represented (Figure 5, Figure 6, Figure 7 and Figure 8). The complete list of the metabolites and the Hotelling-T2 values for each metabolite are reported in Supplementary Table S1.

## 4. Discussion

Rapid seed germination and establishment are vital for survival in natural ecosystems, providing a competitive advantage in securing light, water, and nutrients. Allelopathic species enhance this advantage by releasing chemical compounds that inhibit or delay the germination of nearby plants, thus suppressing potential competitors and enabling their dominance. In our experiments, we examined the effects of VOCs from the fresh leaves of *A. altissima* on the total germination and germination speed of the weed *B. pilosa*. As in other species where *A. altissima* leaf extracts were tested [27], the VOCs released by *A. altissima* significantly reduced the total germination by up to 60% at the highest amount of plant material after eight days of treatment. Notably, concerning the speed of germination, even low concentrations markedly slowed germination speed, reaching 73% inhibition at the highest concentration. In addition, during the experiments we noted that the inhibition of germination observed at the highest concentration was eventually lost, since the seeds germinated after 13 days. Previous experiments already described that the duration of the inhibitory effect of VOCs could be lost over time. Camacho-Coronel et al. [28] found that VOCs, like 4Z-heptenol, farnesene, limonene, and 2E-decenal, inhibited germination rates to less than 25% of control levels after 15 days, although their concentrations decreased over time. This suggests that, while the initial impact of these volatiles is strong, their long-term efficacy may diminish, necessitating further investigation into how these compounds can be effectively utilised over extended periods [28].

Looking at the VOCs characterised during *A. altissima* profiling, several compounds known for their allelopathic activity were identified. For example, Bradow and Connick [29] reported that 2-hexanal was the strongest volatile seed inhibitor released by plant residues, followed by nonanal. Even the compound 3-Hexen-1-ol, a green leaf volatile, plays a significant role in plant defence mechanisms and has been studied for its phytotoxic effects on various plant species. This compound is released rapidly in response to herbivore attacks, serving as a signal for both direct and indirect plant defences. For instance, Liao et al. [30] demonstrated that (Z)-3-hexen-1-ol is emitted within minutes after herbivore damage, indicating its role as a pest-inducible secondary defence compound in plants like tea (Camellia sinensis). Similarly, the emission of (Z)-3-hexen-1-ol has been linked to the activation of defence pathways in other plants, including rice, where it enhances resistance to pests such as the brown planthopper [31]. Besides these compounds, it should be highlighted that the phytotoxic potential of several VOCs reported in Table 2 was largely discussed in the following review [32].

Given that germination involves coordinated activation of multiple metabolic pathways, a metabolomic time-course experiment was conducted to gain further insight into the possible modes of action of these VOCs. Cluster analysis revealed distinct metabolome evolution patterns between treated and untreated seeds, complementing the pathway and enrichment analysis that suggested metabolic alterations. In the untreated seeds, the metabolome developed over time, with the seeds spreading across three clusters corresponding to different developmental stages. The treated seeds, however, showed no progression initially, as T1 and T2 grouped with the control seeds at T0. By T3 and T4, the treated seeds clustered with the control seeds at later stages, indicating a recovery in germination. These results prompted us to identify the pathways affected by treatment and examine the specific metabolites involved.

In this context, the interplay between energy metabolism, amino acid catabolism, and synthesising essential metabolites is crucial for successful seed germination and subsequent seedling development. Both enrichment analysis and pathway analysis highlighted a significant alteration of several pathways involved with germination. For instance, among the affected pathways, glycerolipid metabolism is crucial, providing fatty acids for energy via β-oxidation, essential for early embryo growth [33]. Propanoate metabolism also aids energy production, particularly in seeds utilising fatty acid reserves [33]. Gluconeogenesis converts stored carbohydrates into glucose, supplying energy in early germination [34]. Central to energy production, the TCA cycle and glycolysis convert substrates into energy and essential intermediates, supporting amino acid synthesis [34,35]. Amino acid metabolism, such as valine, leucine, and isoleucine catabolism, supplies energy and carbon skeletons, while arginine and proline metabolism links nitrogen and carbon cycles via TCA cycle intermediates [36,37]. Galactose, starch, and sucrose metabolism provides energy and components for cell expansion [38]. Additionally, glycine, serine, and threonine metabolism contributes precursors necessary for protein synthesis and other metabolic activities [39].

During seed germination, storage compounds, like starch, proteins, and lipids, are broken down to fuel growth. Starch, for example, is converted into simple sugars, like glucose and maltose, providing energy for the embryo. This breakdown is catalysed by enzymes, such as α-amylase, which degrades starch into glucose, a critical energy source and precursor for essential metabolites [40,41]. Disruption of this carbohydrate metabolism by environmental stressors or inhibitors can significantly delay or halt germination [42], and in our experiments, starch and sucrose metabolism resulted among the most impacted pathways. In fact, in the control seeds, the amount of maltose and glucose significantly increased at T1 and T2 and successively dropped at T3 and T4. This suggests that starch was immediately metabolised by the seeds leading to the formation of maltose and glucose, which were immediately metabolised in the late state of the germination (T3–T4). In the treated seeds, maltose content reached the control content only at T2, but then, its amount was significantly higher at T3 and T4, suggesting a lower use of this compound by seed metabolism. Even the amount of glucose was slightly increasing compared to the control in the treated seeds, and only at T4 did its content reach the control level, further suggesting that the treatment slowed down the metabolism. At T1, we observed a decrease in palmitic acid in the treated plants, which later returned to the control level by T2. This finding suggests that, with limited glucose as the primary substrate for glycolysis and energy production, the treated plants may have activated alternative pathways, such as β-oxidation, to generate energy. This hypothesis is further supported by the observed reduction in free phosphate in the treated plants at T2, likely used in ATP production. In contrast, in the control plants, this decrease was already evident at T1. Additionally, changes in glycerolipid and propanoate metabolism align with activating these alternative energy pathways.

Besides starch and sucrose metabolism, galactose metabolism was one of the most affected pathways. Galactose metabolism plays a significant role during seed germination, particularly in the context of the breakdown of raffinose family oligosaccharides (RFOs), which are crucial for providing energy and carbon skeletons necessary for germination [43]. RFOs, such as raffinose and stachyose, are hydrolysed by specific enzymes, releasing galactose and sucrose. This metabolic pathway is essential as it provides energy and influences the osmotic balance within the seed, which is critical during the initial stages of germination [44,45]. In our experiment, the content of raffinose decreased faster in the control seeds than in the treated seeds, and at the same time, the content of galactose increased in the control seeds at T1, probably as a result of raffinose degradation, but no accumulation of sucrose was observed.

In addition to sugars, amino acids also play a pivotal role in seed germination, especially under stressful conditions. The degradation of seed-storage proteins releases amino acids, which can be utilised to synthesise new proteins and other metabolites essential for growth [46,47]. For instance, the GABA shunt pathway, which involves the conversion of glutamate to gamma-aminobutyric acid, is activated during germination under salt stress [48]. In our experiments, we observed a reduction in glutamate and glutamine concentrations in the treated seeds compared to the control and a significant increase in GABA levels at T3 and T4. This suggests that plants respond to VOC-mediated stress by activating this pathway, which helps mitigate oxidative stress and supports the metabolic balance needed for successful germination.

During germination, the release and levels of specific amino acids change significantly. Guo et al. [41] observed that lysine initially rises and then sharply declines after 72 h, suggesting varied amino acid utilisation across germination stages. Similarly, Toyoizumi et al. [49] found notable increases in amino acids, such as GABA, asparagine, glycine, arginine, alanine, proline, valine, isoleucine, leucine, lysine, and phenylalanine, while glutamate and aspartate levels decreased. Besides the previously observed changes induced by the treatment in GABA, glutamate, and glutamine content, the content of the other amino acids significantly changed in the treated seeds. In fact, in the control-developing seed, an accumulation of several amino acids was observed over time. As observed by Guo et al. [41], the lysine content in our experiment initially rose from T0 to T3 before declining. In the treated plants, however, we observed an initial decrease in lysine content during early germination, followed by an increase at T3 and T4, mirroring the accumulation pattern observed in the control seeds at T1 and T2. This suggests that all the metabolic processes connected to lysine accumulation started later in the treated seeds. A similar trend was observed in other amino acids, such as methionine and threonine, which mirrored the control pattern but remained at lower levels until T3, reaching the control values only at T4. Amino acids, like lysine, methionine, and threonine, derived from aspartate, are associated with germination efficiency and subsequent growth [50]. Aspartic acid concentration, which decreased at T1 in the control seeds, showed a reduction in the treated seeds only by T3, suggesting that this delay might result from slower aspartate degradation. This aligns with our findings, where pathway analysis indicated alanine, aspartate, and glutamate metabolism as the most affected pathways.

## 5. Conclusions

This study reveals that *Ailanthus altissima* VOCs not only inhibit *Bidens pilosa* germination but also significantly slow its metabolism. Metabolomic analysis shows delayed starch breakdown, resulting in lower glucose and maltose availability for energy, as well as reduced glycolysis and TCA cycle activity, suggesting limited energy production. Amino acid metabolism, particularly involving lysine and threonine, also slowed, delaying critical protein synthesis needed for growth.

This metabolic deceleration likely weakens *B. pilosa*’s competitive edge during early growth stages, thus enhancing *A. altissima*’s invasive advantage by suppressing nearby competitors. These findings emphasise the potential of VOC-mediated allelopathy as a mechanism for *A. altissima*’s ecological dominance and invite further exploration into VOC applications for invasive species control.

## Figures and Tables

**Table 1:** Chemical characterisation of *Ailanthus altissima* volatile organic compounds.

RT	RI	Metabolite Name	SubClass	RAP%
2.517	681.83	2-ethyl-furan	Furanoid	0.45 (±0.08)
2.867	706.23	2-Methyl-1-butanol	Alcohols and polyols	0.57 (±0.17)
2.99	714.66	(E)-2-methyl-2-Butenal	Carbonyl compounds	0.19 (±0.10)
3.446	746.61	1-Pentanol	Alcohols and polyols	0.12 (±0.04)
3.594	756.92	1-Penten-3-ol, 2-methyl-	Alcohols and polyols	0.41 (±0.06)
3.684	763.25	Methyl α-methylbutanoate	Fatty acid esters	0.11 (±0.04)
4.192	798.74	3-Hexenal	Carbonyl compounds	1.11 (±1.15)
4.23	800.16	Heptane, 2,4-dimethyl-	Alkanes	0.77 (±0.04)
4.244	800.81	Hexanal	Carbonyl compounds	0.57 (±0.28)
4.435	805.44	3-Octene, (Z)-	Unsaturaed aliphatic hydrocarbons	0.28 (±0.27)
5.332	826.58	3-Hexene, 1-methoxy-, (Z)-	Ethers	0.24 (±0.05)
6.18	846.69	Butanoic acid, 2-methyl-, ethyl ester	Fatty acid esters	0.30 (±0.23)
6.224	847.76	3-Hexen-1-ol, (E)-	Fatty alcohols	0.60 (±0.22)
6.395	851.78	3-Hexen-1-ol, (Z)-	Fatty alcohols	47.52 (±12.25)
6.573	856.22	4-Hexen-1-ol, (Z)-	Fatty alcohols	10.73 (±1.72)
6.976	865.51	2-Hexen-1-ol, (Z)-	Fatty alcohols	1.52 (±1.07)
7.63	881.05	1-Butanol, 2-methyl-, acetate	Carboxylic acids derivatives	0.38 (±0.17)
8.423	899.9	4,4-Dimethyl octane	Alkanes	0.12 (±0.05)
9.052	921	Amyl acetate	Carboxylic acids derivatives	0.11 (±0.04)
9.349	931.05	Dimethylallyl acetate	Carboxylic acids derivatives	0.32 (±0.26)
9.541	937.55	alpha-Pinene	Monoterpenoids	1.49 (±0.22)
10.74	978.03	β-Thujene	Monoterpenoids	0.13 (±0.01)
11.205	993.75	p-Mentha-1(7),8-diene	Monoterpenoids	1.66 (±1.26)
11.54	1009.79	3-Methyl-4-penten-1-ol acetate	Carboxylic acids derivatives	58.58 (±47.89)
11.659	1017.68	Hexyl acetate	Carboxylic acids derivatives	4.33 (±1.23)
11.711	1020.99	2-Hexen-1-yl-acetate	Carboxylic acids derivatives	3.50 (±2.65)
11.872	1032.82	3-Carene	Monoterpenoids	0.31 (±0.20)
11.892	1032.82	L-Limonene	Monoterpenoids	0.76 (±0.31)
12.064	1044.02	1,4-p-Menthadien-7-ol	Alcohols and polyols	0.28 (±0.03)
12.083	1045.27	o-Cresol	Cresols	0.18 (±0.16)
12.23	1054.92	cis-β-Ocimene	Monoterpenoids	1.03 (±0.58)
12.345	1062.39	Artemisia alcohol	Alcohols and polyols	0.19 (±0.20)
12.445	1068.61	Artemesia ketone	Carbonyl compounds	0.55 (±0.12)
12.792	1091.65	Isoterpinolene	Monoterpenoids	0.49 (±0.20)
12.935	1101.41	Linalyl formate	Monoterpenoids	0.39 (±0.26)
12.987	1106.3	Nonanal	Carbonyl compounds	0.21 (±0.09)
13.134	1119.96	Perillene	Monoterpenoids	8.25 (±5.77)
13.43	1147.7	(Z)-3-Hexenyl butanoate	Fatty acid esters	0.24 (±0.10)
13.678	1170.83	α-Acetoxytoluene	Benzyloxycarbonyls	0.20 (±0.23)
13.768	1179.3	Ethyl benzoate	Benzoic acids and derivatives	0.15 (±0.03)
13.864	1188.19	(E)-2,6-Dimethyl-3,7-octadien-2,6-diol	Alcohols and polyols	0.15 (±0.13)
14.022	1203.86	Methyl salicylate	Benzoic acids and derivatives	7.92 (±8.17)
14.284	1235.04	cis-3-Hexenyl valerate	Fatty acid esters	2.84 (±1.02)
14.319	1239.2	Hexyl 2-methylbutyrate	Fatty acid esters	0.19 (±0.02)
14.797	1296.07	2-Undecanone	Carbonyl compounds	0.04 (±0.05)
14.84	1301.33	Dihydroedulan II	Sesquiterpenoids	0.05 (±0.01)
15.192	1348.96	α-Longipinene	Sesquiterpenoids	0.67 (±0.60)
15.297	1363.12	Copaene	Sesquiterpenoids	11.52 (±9.81)
15.353	1370.63	Longicyclene	Sesquiterpenoids	0.48 (±0.35)

RT: retention time; RI: retention index; RAP%: area percentage of a specific metabolite calculated on the total area of the metabolites identified. The information concerning the peak area, spectra total score similarity (% of match with the library), retention index (RI) similarity, S/N ratio, and EI spectrum are reported in Supplementary Table S1.

**Table 2:** Result from “Pathway Analysis” (topology + enrichment analysis) carried out on the metabolite identified in *B. pilosa* seeds during the germination process (T0–T4) in response to the VOCs produced by *A. altissima* (0 g and 150 g).

Pathway	Total Cmpd	Hits	Raw *p*	FDR	Impact
Alanine aspartate and glutamate metabolism	22	7	3.37 × 10^−11^	9.26 × 10^−10^	0.77698
Galactose metabolism	27	9	2.02 × 10^−10^	2.77 × 10^−9^	0.64052
Starch and sucrose metabolism	22	7	3.83 × 10^−12^	2.11 × 10^−10^	0.60856
Isoquinoline alkaloid biosynthesis	6	1	4.33 × 10^−6^	4.98 × 10^−6^	0.5
Phenylalanine metabolism	12	1	2.16 × 10^−6^	3.49 × 10^−6^	0.42308
Arginine biosynthesis	18	6	8.27 × 10^−9^	4.13 × 10^−8^	0.36117
Glycine serine and threonine metabolism	33	4	4.92 × 10^−9^	3.01 × 10^−8^	0.32415
Fructose and mannose metabolism	18	2	1.12 × 10^−6^	2.00 × 10^−6^	0.29099
Arginine and proline metabolism	32	5	7.14 × 10^−9^	3.93 × 10^−8^	0.2634
Beta-alanine metabolism	18	2	1.04 × 10^−8^	4.42 × 10^−8^	0.25397
Citrate cycle (TCA cycle)	20	4	1.94 × 10^−10^	2.77 × 10^−9^	0.21839
Amino sugar and nucleotide sugar metabolism	52	3	2.36 × 10^−7^	6.50 × 10^−7^	0.2096
Tryptophan metabolism	29	1	2.54 × 10^−7^	6.64 × 10^−7^	0.20611
Tyrosine metabolism	17	2	8.72 × 10^−7^	1.81 × 10^−6^	0.20112

Total Cmpd: the total number of compounds in the pathway; Hits: the matched number from the uploaded data; Raw *p*: the original *p*-value; FDR: the false discovery rate applied to the nominal *p*-values to control for false-positive findings; Impact: the pathway impact value calculated from pathway topology analysis.

## Data Availability

The data presented in this study are available in the article and Supplementary Material.

## Supplementary Materials

The following supporting information can be downloaded at: https://www.mdpi.com/article/10.3390/metabo15010012/s1, Table S1: Volatilome and untargeted-metabolomics raw data and statistical analysis.
